# Derinat^®^ has an immunomodulatory and anti-inflammatory effect on the model of acute lung injury in male SD rats

**DOI:** 10.3389/fphar.2022.1111340

**Published:** 2022-12-30

**Authors:** Yulia A. Palikova, Victor A. Palikov, Nadezhda I. Novikova, Gulsara A. Slashcheva, Ekaterina A. Rasskazova, Elena A. Tukhovskaya, Alexey V. Danilkovich, Igor A. Dyachenko, Alexey A. Belogurov Jr., Anna A. Kudriaeva, Daniil Y Bugrimov, Olga N. Krasnorutskaya, Arkady N. Murashev

**Affiliations:** ^1^ Branch of Shemyakin-Ovchinnikov Institute of Bioorganic Chemistry, Russian Academy of Sciences (BIBCh RAS), 6 Prospekt Nauki, Pushchino, Russia; ^2^ Shemyakin-Ovchinnikov Institute of Bioorganic Chemistry, Russian Academy of Sciences (IBCh RAS), 16/10 Miklukho-Maklay Str, Moscow, Russia; ^3^ Department of Biological Chemistry, Evdokimov Moscow State University of Medicine and Dentistry, Moscow, Russia; ^4^ Voronezh State Medical University Named After N. N. Burdenko, 10 Studencheskaya Str, Voronezh, Russia

**Keywords:** Derinat^®^ (deoxyribonucleic acid sodium salt), acute lung injury (ALI), cytokines, spirometry, rats

## Abstract

To simulate acute lung injury (ALI) in SD male rats they we administered intratracheally with lipopolysaccharide (LPS) followed by hyperventilation of the lungs (HVL), which lead to functional changes in the respiratory system and an increase in the blood serum concentration of inflammatory cytokines. LPS + HVL after 4 h lead to pronounced histological signs of lung damage. We have studied the effectiveness of Derinat^®^ when administered intramuscularly at dose of 7.5 mg/kg for 8 days in the ALI model. Derinat^®^ administration lead to an increase in the concentration of most of the studied cytokines in a day. In the ALI model the administration of Derinat^®^ returned the concentration of cytokines to its original values already 48 h after LPS + HVL, and also normalized the parameters of pulmonary respiration in comparison with animals without treatment. By the eighth day after LPS + HVL, respiratory parameters and cytokine levels, as well as biochemical and hematological parameters did not differ between groups, while histological signs of residual effects of lung damage were found in all animals, and were more pronounced in Derinat^®^ group, which may indicate stimulation of the local immune response. Thus, the administration of Derinat^®^ stimulates the immune response, has a pronounced protective effect against cytokinemia and respiratory failure caused by ALI, has immunomodulatory effect, and also stimulates a local immune response in lung tissues. Thus, Derinat^®^ is a promising treatment for ALI.

## 1 Introduction

Acute lung injury (ALI) is a syndrome characterized by acute inflammation and tissue damage that interferes with normal gas exchange in the lungs. Patients with ALI have severe hypoxemia and radiographic evidence of bilateral pulmonary edema. Despite^®^ the recent decline in ALI mortality, ALI is still a significant factor in morbidity and mortality among critically ill patients (Mowery Nathan et al., 2020). Mortality from ALI for some groups of patients is high - up to 60%, and on average remains at 40% (Bellani et al., 2016). Inflammatory lung diseases, including pneumonia, ALI, acute respiratory distress syndrome, and chronic obstructive pulmonary disease, cause significant morbidity and mortality worldwide (Marco et al., 2007). The main causes of ALI are infectious etiologies such as sepsis and pneumonia (Ware and Mattay, 2000). Acute respiratory distress syndrome, a clinically important complication of ALI in humans, is responsible for mortality in 40% of critically ill patients (Rubenfeld et al., 2005; Marco et al., 2007). The physiological sign of ALI is a violation of the alveolar-capillary membrane barrier, leading to the development of non-cardiogenic pulmonary edema, in which the protein exudate fills the alveolar spaces, impairs gas exchange and accelerates the development of respiratory failure (Guidot et al., 2006; Marco et al., 2007).

Despite years of research, few therapeutic strategies have emerged for the treatment of clinical ALI. Diagnosis of ALI can be difficult, which makes it difficult to accurately and quickly determine the etiology for the development of targeted therapeutic interventions. Depending on the severity of ALI, different therapeutic approaches are used. Primarily, therapy is aimed at combating the underlying disease/cause of lung damage, which depends on the etiology and includes infection control, surgical treatment, increased oxygenation, dehydration of the lungs, provision of nutrients, treatment of shock caused by an increase in cytokine levels (Michael et al., 1998; Gerlach et al., 1999; Lundin et al., 1999; Protti et al., 2000; Kallet, 2004; Meade et al., 2008; Frat et al., 2015; Stéphan et al., 2015; Beitler et al., 2016; McClave et al., 2016; Vieillard-Baron et al., 2016; Combes et al., 2018; Singer et al., 2019). If the condition is critical, the therapy of choice is respiratory therapy, namely tracheal intubation and mechanical ventilation (ALI), which, however, may not be effective in hyperoxia, especially in the presence of concomitant aggravating conditions such as heart attack, stroke, circulatory arrest (Damiani et al., 2014; Elmer et al., 2015; Hofmann et al., 2017; Roffe et al., 2017; Aggarwal et al., 2018; Page et al., 2018). Respiratory support is also important, requiring expensive, modern ventilators (Chatburn, 2003; Tobin, 2013). It is extremely important to develop new effective and safe therapeutic schemes aimed at the mechanisms of attraction and accumulation of inflammatory cells.

At the tissue level, ALI in humans is characterized by neutrophilic alveolitis, damage to the alveolar epithelium and endothelium, hyaline membrane formation, and microvascular thrombi. The fundamental mechanisms underlying ALI remain poorly understood (Hong et al., 2010; Hoegl et al., 2018). It is known that with the onset and development of an inflammatory process, including in the lung tissue, there is an increase in the production of cytokines and chemokines. Due to disruption of the integrity of the alveolar-capillary barrier, immune cells migrate into lung tissue (Cross and Mattay, 2011; Bhargava and Wendt, 2012). Cells of the immune system, such as macrophages, neutrophils, dendritic cells, natural killer cells, are able to secrete various cytokines. However, cytokines are most actively synthesized by macrophages and T-helper cells. In particular, activated macrophages and epithelial cells produce inflammatory mediators such as tumor necrosis factor alpha (TNF-α) and interleukin-1 beta (IL-1β), which in turn induce neutrophil recruitment and release of additional cytokines, including IL-6 (Rittirsch et al., 2008; Chimenti et al., 2020; Domscheit et al., 2020). The cytokine surge (increased levels of cytokines) during the inflammatory process can be modeled *in vitro* and *in vivo* using various stimuli (Domscheit et al., 2020).

LPS endotoxin, which is part of the outer membrane of Gram-negative bacteria, is well known for its ability to induce lung inflammation, and experimental administration of LPS, both systemically and intratracheally, is a standard stimulus for inducing inflammation. LPS induces activation of innate immunity through CD14 and Toll-like receptor (TLR) 4, which leads to the induction of an inflammatory response by activating the transcription nuclear factor-κB (NF-κB) (Rittirsch et al., 2008). Animal models of ALI using LPS are characterized by bronchoalveolar neutrophilia and elevated cytokines (Jean-Damien et al., 2001; Switalla et al., 2010). In experimental ALI, the lung parenchyma is damaged due to the generation and release of proteases, reactive oxygen species, nitrogen produced by activated lung macrophages and neutrophils in the interstitial and alveolar spaces (Hoegl et al., 2018). Animal models are an important tool in testing the hypothesis of the underlying molecular mechanisms of ALI and can serve as a bridge between drug development and its introduction into clinical practice, as well as in the development of new therapeutic strategies. The cytokines TNF-α, IL-1β, IL-8, and an IL-1 receptor antagonist may be considered therapeutic targets for LPS-induced ALI, and many treatments have been shown to inhibit cytokine expression in the lungs (Barnes, 2006; Hong et al., 2010; Hoegl et al., 2018; Song et al., 2021).

Currently, corticosteroids are used for ALI treatment, which have a number of positive properties, such as anti-inflammatory and decongestant. The mechanism of action of corticosteroids associated with the activation of cytosolic glucocorticoid receptors, which, when bound to glucocorticoids, migrate to the cell nucleus, where they activate the transcription of anti-inflammatory proteins such as IL-10, annexin 1, an inhibitor of NF-κB, *etc.*, as well as and other regulatory proteins that affect metabolism (Rhen and Cidlowski, 2005; Stahn and Buttgereit 2008), and also inhibits transcription of inflammatory transcription factor proteins such as NF-κB andactivatorprotein-1 (AP-1) The described processes are the main mechanism of anti-inflammatory and immunosuppressive effects causing suppression of the synthesis of pro-inflammatory cytokines such as L-1, TNFα, interferon IFNγ, etc (Rogatsky and Ivashkiv, 2003).

The use of immunomodulatory drugs seems promising for the treatment of ALI. Derinat^®^ (sodium salt of deoxyribonucleic acid) is a polydeoxynucleotide of eukaryotic origin with a molecular weight of up to 500 kDa, obtained from salmon and/or sturgeon milk. The Derinat^®^ molecule consists of an average of 750 base pairs. Derinat^®^ consists of DNA fragments ending in 5–25% of cases with unmethylated CpG motifs. Unmethylated CpG DNA motifs are ligands for TLR9 membrane proteins belonging to the group of Toll-like receptors. It is known that recognition of unmethylated CpG motifs in bacterial and viral DNA triggers the response of the innate immune system. During the preparation of the drug, DNA molecules contained in salmon sperm homogenates are fragmented by ultrasonic treatment, which leads to the exposure of CpG motifs, making them available for interaction with cells expressing the TLR9 receptor (Guidot et al., 2006). The receptor TLR9 and NF-kB and MyD88, which are widespread in the body, are a dependent way of transmitting a stimulating signal to immunocompetent cells, which determines a wide range of therapeutic possibilities of the drug. Carcinogenic effects on the body (Marco et al., 2007; Filatov et al., 2013; Paiva end Bozza, 2013). Clinical studies have shown that Derinat^®^ has an immunomodulatory effect in the treatment of sepsis, inflammatory conditions and ulcers (Gromov and Pivovarova, 2002; Okayama, 2005; Handa et al., 2010; Wang et al., 2010; Gora et al., 2011; Hsu et al., 2015; Liu et al., 2018). For example, in a study of rheumatoid arthritis, Derinat^®^ was found to suppress tumor necrosis factor alpha (TNF-α) and accelerate blast transformation of lymphocytes in rats (Zemskov et al., 2011). Our goal was to study the effect of Derinat^®^ on the induction and development of the inflammatory process in the LPS-induced ALI model. In particular, we set the task of evaluating the effect of the drug on the dynamics of a wide range, as well as the physiological parameters of respiration in rats. In order to bring the manifestation of ALI in animals closer to the clinical scenario, in our study we used a two-hit model, including intratracheal administration of LPS and subsequent hyperventilation of the lungs (Mangushev et al., 2008). We assume that the double exposure method will allow us to evaluate in detail the effect of the drug and more reliably predict the effectiveness of Derinat^®^ in the treatment of ALI.

## 2 Materials and methods

### 2.1 Animals

23 mature specific pathogen free (SPF) male SD rats, 10–12 weeks old were obtained from the Pushchino nursery of laboratory animals (Pushchino, Russia). All procedures and manipulations with animals were approved by the Committee for Control over Care and Use of Laboratory Animals of BIBCh RAS (IACUC) (protocol number 711/19 from 23.08.2019) and were carried out in accordance with the EU Directive 2010/63/EU. After receiving from the nursery, the animals underwent adaptation within 7 days. During this period, the animals were monitored for signs of deviation in health status. Animals without signs of health deviations were selected for the experiment (clinical examination was done). Animals were divided into groups using the principle of randomization, using body weight as a criterion, so that the average body weight of animals by the first day of administration did not differ statistically between groups. Each animal was assigned an individual number, according to which the animal was marked with a puncture of the auricle. The cell label indicated the group, animal number, label, study code, head full name, IACUC protocol number, group color code. During the study, the animals were kept under controlled environmental conditions in a barrier zone with a “clean” and “dirty” corridor system with controlled environmental conditions (temperature 20°C–24°C, relative humidity 30–55%, 12-h light cycle). 08:00–20:00 - “day”, 20:00–08:00 - “night”, 10-fold change in air volume in the room per hour). SNIFF RI/M-H V1534-30 complete granular rodent food was autoclaved and fed *ad libitum*.

#### 2.1.1 Study design

**FIGURE 1:**
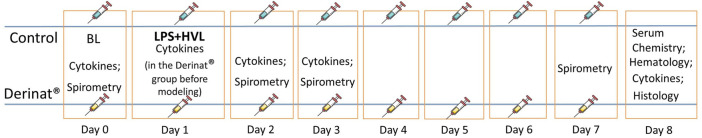
Experiment scheme.

To verify the ALI model, before the start of the study, three animals were injected with LPS followed by HVL, after which they were euthanized after 4 h and the lungs were taken for histological confirmation of the development of ALI. 20 male SD rats were divided into two groups of 10 animals. Group 1 (control) was injected intramuscularly with saline in a volume of 0.5 ml/kg on days 0, 2, 3, 4, 5, 6, seven of the study. Group 2 (Derinat^®^) received Derinat^®^ at a dose of 7.5 mg/kg intramuscularly in a volume of 0.5 ml/kg on days 0, 2, 3, 4, 5, 6, 7 of the study. The dose of Derinat at 7.5 mg/kg was determined based on the therapeutic dose for humans given in the instructions for use (http://www.derinat.com/en/catalog/items/103-derinat-solution-for-intramuscular-injection/). The rat dose was converted according to the guidelines (https://www.fda.gov/media/72309/download), i.e. 75 mg/65 kg (mean human weight) * 6.2 (conversion factor per rat) = 7.2 mg/kg. We have rounded the dose up to 7.5 mg/kg, as in the study by Kruglov et al., 2015. Course administration for 8 days was also based on the instructions for use of the drug in inflammatory diseases (http://www.derinat.com/en/catalog/items/103-derinat-solution-for-intramuscular-injection/), suggesting a duration of therapy up to 10 days. ALI was simulated by a single injection of LSP on the first day of the study (the scheme of the experiment is shown in Figure 1). To assess the functional state of the lungs, respiratory parameters were measured (spirometry) on days 0, 2, 3, and 7 (the day before euthanasia) of the study. Animals of both groups were bled before ALI modeling, and then from animals from group 1 on days 2, 3 and 8 after ALI modeling, and from animals from group 2 (Derinat^®^) - 1 day after the preventive administration of Derinat to assess the effect of the drug itself on cytokine parameters, and then on days 2, 3 and 8 after ALI modeling. Blood were taken from all animals before ALI modeling, and then from animals of group 1 on days 2, 3 and 8 after ALI modeling, and from animals of group 2 (Derinat^®^) - 1 day after the preventive administration of Derinat^®^ to assess the effect of the drug for cytokine indices, and then on days 2, 3 and 8 after ALI modeling. The concentration of cytokines and biochemical parameters were analyzed in the blood. At the end of the study, the animals were euthanized and the lungs were taken for histological analysis.

### 2.2 ALI modeling

ALI modeling was performed for all animals under general anesthesia (Zoletil^®^/Xyla^®^). As factors causing the development of ALI in rats, a combination of two effects was used - intratracheal administration of LPS and subsequent hyperventilation of the lungs (HVL): Intratracheal administration was performed on an anesthetized animal, fixed on the operating table in the supine position, fixing it by the paws and upper incisors. The lower jaw was retracted and the oral cavity was opened. A metal probe with a ball at the end was inserted over the surface of the tongue into the oral cavity. Having overcome the resistance of the vocal cords (on exhalation), the probe was inserted into the trachea. A thin catheter was inserted into the opening of the probe and introduced to a pre-measured depth. A syringe with LPS at a dose of 1 mg/kg (lot # 019M4009V, Sigma Aldrich, USA) was connected to the catheter, and, while holding the animal in a vertical position, LPS was slowly injected in a volume of 0.5 ml/kg Which was performed in all animals 20 min after intratracheal administration of LPS. The animal was connected intratracheally to an Ugo Basile 7025 ventilator *via* a soft catheter tube. For hyperventilation, an increased tidal volume of the lungs was used - 30 ml/kg, respiratory rate 60 times/min, exposure time 20 min.

### 2.3 Test item and vehicle

Derinat^®^ (Series 30901017, LLC FZ Immunnolex, Russia) and the carrier - physiological saline (0.9% sodium chloride for infusion) were administered intramuscularly on study days: 0, 2, 3, 4, 5, 6, 7. Derinat^®^ was administered intramuscularly at a dose of 7.5 mg/kg, 0.5 ml/kg. Physiological saline was administered intramuscularly in a volume of 0.5 ml/kg.

### 2.4 Assessment of the respiratory system parameters

Spirometry was performed on day 0 of the study before ALI modeling, on day 2 - 1 day after modeling, on day 3, and on the eve of necropsy - on day 7. Recording of respiratory parameters was carried out on the FE141 Spirometer device (Ser. # FE141-0300) on the PowerLab 8/35 (Ser. # 3508–1090) computer system (ADInstruments Pty Ltd, Australia). The following parameters were recorded: respiratory rate (times/min), tidal volume L), maximum expiratory flow (L/s).

### 2.5 Taking blood samples for cytokine analysis

For the analysis of cytokine concentrations, plasma of animal blood samples was used, which were collected at the following time points:

Day 0—before interventions (groups 1, 2)
Day 1—a day after the prophylactic administration of Derinat^®^, before modeling (only group 2)
Day 2—a day after the modeling (groups 1, 2)
Day 3—a day after modeling (groups 1, 2)
Day 8—blood samples were obtained during necropsy after anesthesia (groups 1, 2)

Blood was taken from the lateral vein of the tail using a catheter needle (24G, 0.7 × 19 mm). For analysis, blood samples (∼0.4 ml) were placed in Microvette^®^ tubes containing K3EDTA. After that, the tubes with blood were centrifuged (1600 g, 4°C, 15 min) and plasma was collected. Plasma was frozen and stored at -20°C until the analysis of the content of cytokines in blood plasma. The Bio-Plex Pro™ Rat Cytokine 23-Plex kit (Cat. #12005641) was used to measure the content of cytokines in blood plasma, including the determination of the following cytokines: G-CSF, GM-CSF, GRO/KC, IFN-γ, IL-1α, IL-1β, IL -2, IL-4, IL-5, IL-6, IL-7, IL-10, IL-12 (p70), IL-13, IL-17A, IL-18, M-CSF, MCP-1, MIP-1α, MIP-3α, RANTES, TNF-α, VEGF. Bio-Plex Pro Assays are immunoassays formatted on magnetic beads. The assay principle is similar to that of a sandwich ELISA. Capture antibodies directed against the desired biomarker are covalently coupled to the beads. Coupled beads react with the sample containing the biomarker of interest. After a series of washes to remove unbound protein, a biotinylated detection antibody is added to create a sandwich complex. The final detection complex is formed with the addition of streptavidin-phycoerythrin (SA-PE) conjugate. Phycoerythrin serves as a fluorescent indicator or reporter.

### 2.6 Euthanasia and necropsy

Animals were euthanized by anesthesia (Zoletil^®^+Xyla^®^, 30 mg + 10 mg/kg) followed by terminal blood sampling from the inferior vena cava to study the cytokine profile. During necropsy, a portion of the lungs was taken and fixed in 10% neutral formalin for microscopic analysis. Lung sampling was performed by filling the lungs with a formalin solution.

### 2.7 Hematological analysis

Hematological analysis was performed on the eighth day of the study. Blood samples of 1 ml were placed in microtubes with K3EDTA. Hematological analysis was carried out using a Mythic 18 hematological analyzer (Ser. # 18100313007161) with a veterinary program (C2 DIAGNOSTICS S.A, France). The following parameters were measured: red blood cell count, hemoglobin level, hematocrit, white blood cell count, lymphocyte count, monocyte count, granulocyte count, mean erythrocyte hemoglobin content, mean erythrocyte hemoglobin concentration, mean erythrocyte volume, erythrocyte distribution width by volume–coefficient of variation, erythrocyte distribution width by volume–standard deviation, platelet count, mean platelet volume, thrombocrit, platelet volume distribution breadth - coefficient of variation.

### 2.8 Biochemical analysis

Biochemical analysis was performed on the eighth day of the study. The blood was placed in a test tube without anticoagulant and left for 50 min to clot. Then centrifuged (1600 g, 4°C, 15 min) to separate the serum. The following indicators were determined in blood serum: alanine aminotransferase, aspartate aminotransferase, total protein, albumin, globulins, creatinine, triglycerides. Measurements were carried out on a Sapphire-400 (Ser. # 2708820712) automatic biochemical analyzer (Tokyo Boeki LTD., Japan) using Randox GB reagent kits appropriate for each parameter.

### 2.9 Histological analysis

Biomaterial samples were fixed in 10% neutral formalin solution (at least 2 days, room temperature), washed in running tap water, dehydrated in ascending concentration alcohols (within 24 h at room temperature), and embedded in paraffin (1 hour at 58°C). Paraffin sections three to five μm thick, stained with hematoxylin and eosin, were examined using conventional light microscopy on an AxioScope.A1 microscope (Carl Zeiss, Germany). To assess the severity of histological changes, a scoring scale was used (Mann et al., 2012).

### 2.10 Statistical analysis

Statistical data processing was performed using Statistica v.7.1 software for Windows. Repeated measurements of the cytokine profile were analyzed using the repeated measures ANOVA two analysis of variance. Pairwise comparison of cytokine concentrations in the two groups (Control group vs Derinat^®^ group) on the same day of the study was performed using the non-parametric Mann-Whitney U test.

## 3 Results

### 3.1 Assessment of breathing parameters

**FIGURE 2:**
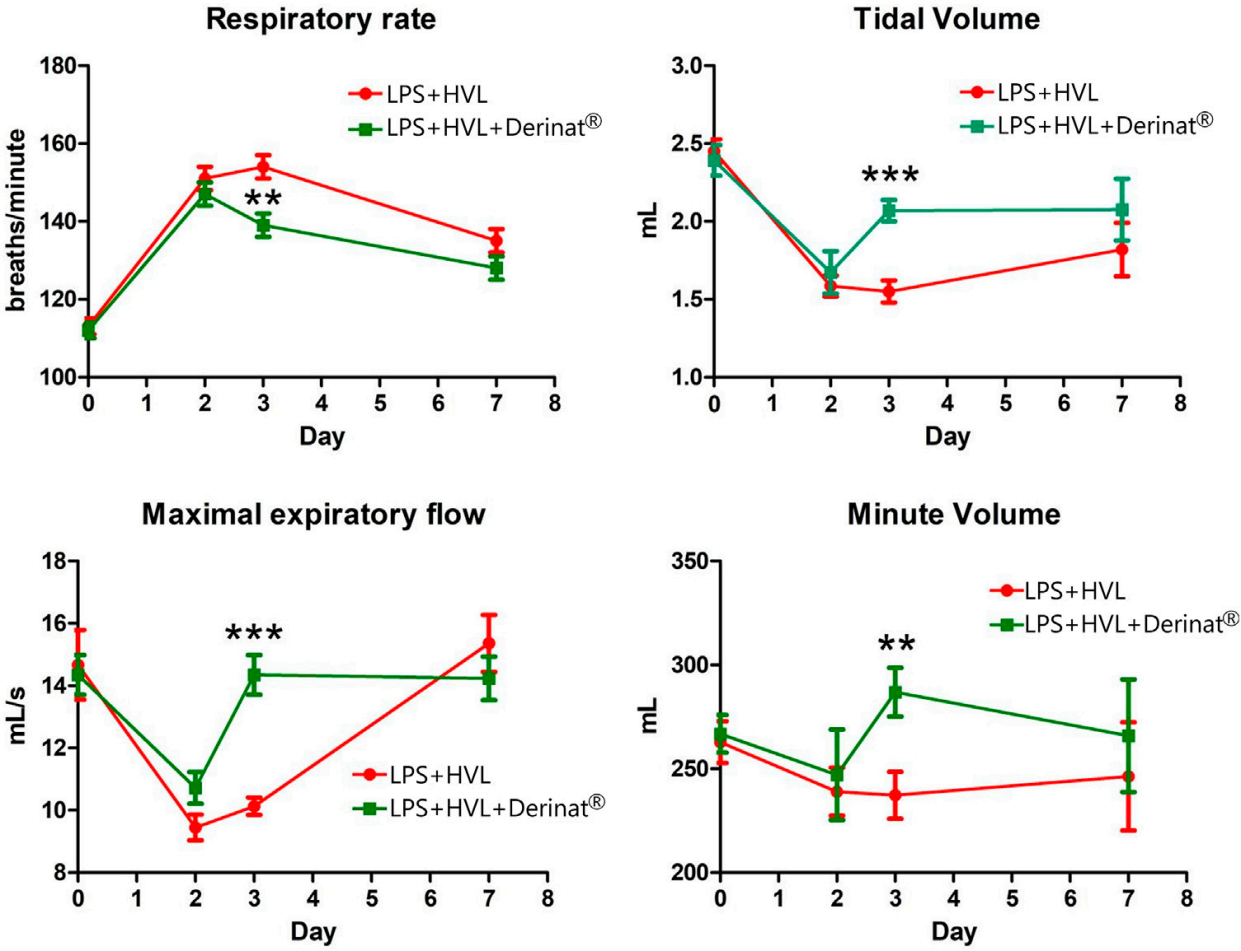
Dynamics of functional changes in the respiratory system of rats (minute volume of respiration; volume of respiration; respiratory rate; maximum expiratory flow) with the simulation of APL on the background of the introduction of saline or Derinat^®^. * - *p* < 0.05, ** - *p* < 0.01, *** - *p* < 0.001 relative to the control group without ALI modeling according to the Mann-Whitney U test.

A single intratracheal administration of LPS + HVL led to changes in the respiratory system of animals, namely, an increase in the frequency of respiratory movements by 34%, tidal volume by 35%, minute respiratory volume by 9%, while the expiratory flow rate was reduced by 36% indicating a decrease in lung capacity. Figure 2 shows that the use of Derinat^®^ allows animals to fully restore respiratory function already 48 h after ALI modeling, in contrast to animals that did not receive therapy.

### 3.2 Derinat^®^ changes the levels of cytokines in the blood plasma of rats with ALI modeling

**FIGURE 3:**
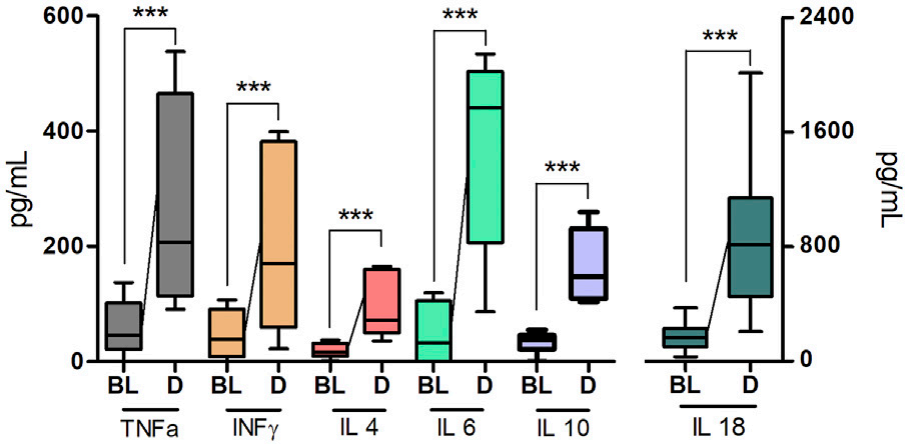
The concentration of some cytokines (TNFα, IFNγ, IL-4, IL-6, IL-10, IL-18) 24 h after a single intramuscular injection of Derinat^®^ at a dose of 7.5 mg/kg.

**FIGURE 4:**
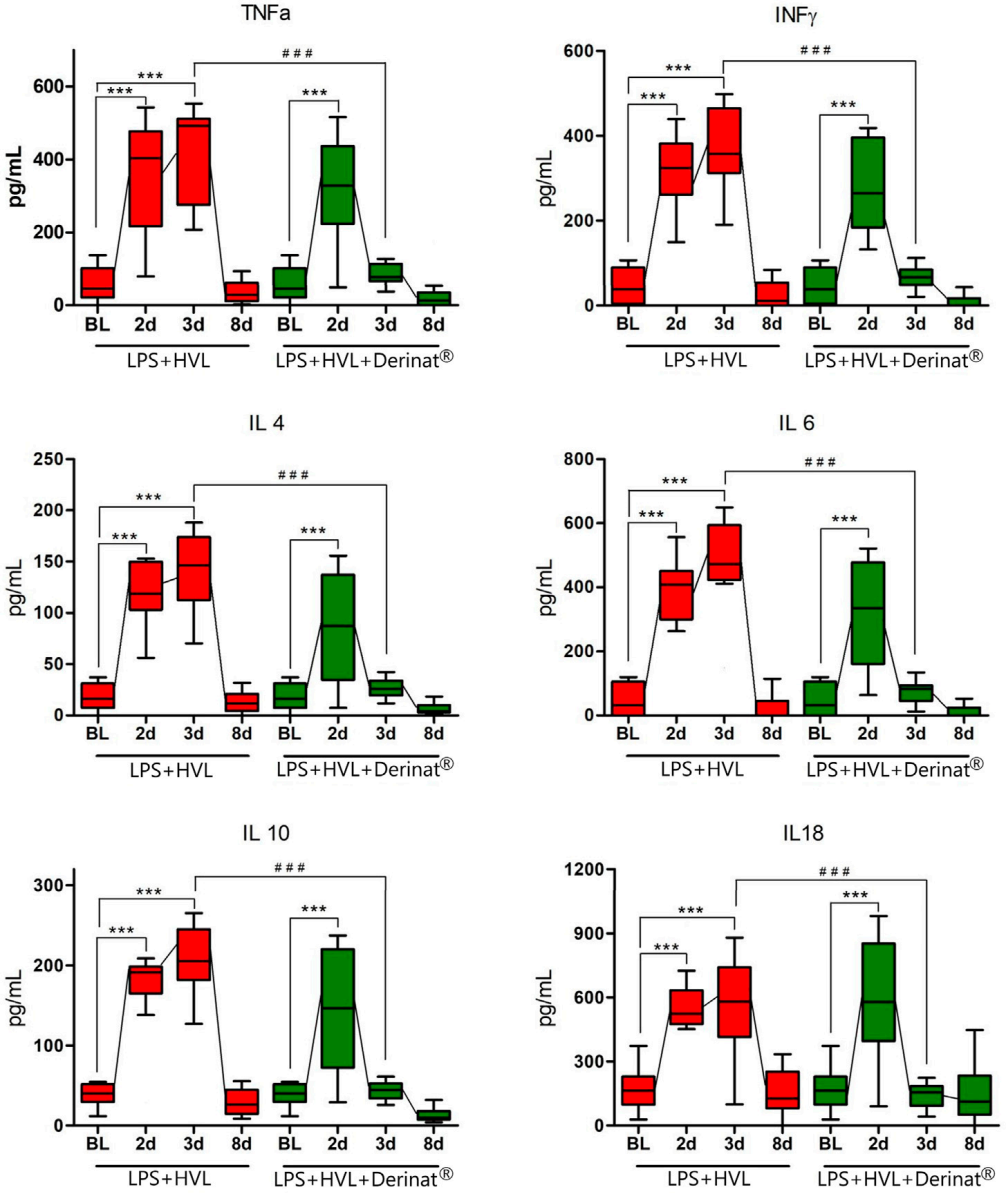
Dynamics of changes in the concentration of some cytokines (TNFα, IFNγ, IL-4, IL-6, IL-10, IL-18) in the blood plasma of SD rats on the background of the introduction of saline or Derinat^®^. BL–Baseline (value prior ALI modeling); 2d, 3d, 8d after ALI simulation. #*p* ≤ 0.05, ##*p* ≤ 0.01, ###*p* ≤ 0.001 relative to BL (Mann-Whitney U test) *p ≤ 0.05, **p ≤ 0.01, ***p ≤ 0.001 relative to previous internal value groups according to the repeated measures ANOVA 2

A single intramuscular injection of Derinat^®^ at a dose of 7.5 mg/kg to intact animals causes an increase in the concentration of most of the studied cytokines several times in blood plasma 24 h after its administration (Figure 3; Table 1). Intratracheal administration of LPS and hyperventilation of the lungs provoked a surge of cytokines in intact animals after 24 h, similar in nature to the administration of Derinat^®^ (Figure 4; Table 1). Modeling of ALI in animals treated with Derinat^®^ the day before did not lead to additional changes in the cytokine level. 48 h after ALI modeling, in animals receiving Derinat^®^, the level of cytokines reached the values of intact animals. Animals without therapy, 48 h after the simulation, still had a significantly increased level of cytokines (Figure 4; Table 1). On the eighth day of the study, the concentration of cytokines in the blood plasma in both groups returned to the baseline (Table 1).

**TABLE 1:** The concentration of cytokines in the blood of SD rats in dynamics before and after ALI simulation.

Cytokine, pg/mL	BL *n* = 18^§^	LPS + HVL			LPS + HVL + Derinat®			
		Day1	Day2	Day 8	Derinat®	Day1	Day2	Day 8
		+LPS *n* = 10	+LPS *n* = 10	+LPS *n* = 10	*n* = 10	+LPS *n* = 10	+LPS *n* = 10	+LPS *n* = 10
TNFa	56,3 ± 10,5	361,9 ± 47,2	426,3 ± 42,6	36,1 ± 9,8	251,7 ± 56,6	290,6 ± 54,2	80,2 ± 9,5	31,7 ± 16,7
		***	***		***	***	###	
IFNγ	42,5 ± 9,0	281,7 ± 40,7	349,0 ± 40,6	25,3 ± 9,7	187,2 ± 49,8	222,6 ± 47, 8	67,0 ± 8,5	9,0 ± 5,0
		***	***		^**^	^***^	###	
IL-4	18,3 ± 2,9	110,2 ± 13,2	142,1 ± 12,2	12,7 ± 3,3	87,6 ± 18,2	88,0 ± 17,2	26,6 ± 3,0	6,7 ± 1,8
		***	***		^***^	***	###	
IL-6	42,9 ± 10,9	323,4 ± 54,1	422,9 ± 64,2	24,0 ± 12,2	187,2 ± 71,9	250,4 ± 62,8	72,3 ± 11,6	6,7 ± 5,3
		***	***			^**^	###	
IL-10	32,9 ± 4,1	171,7 ± 17,0	206,5 ± 13,1	26,1 ± 5,8	156,6 ± 21,1	144,6 ± 23,3	43,1 ± 3,6	13,4 ± 2,9
		***	***		***	***	###	
IL-18	171,3 ± 22,2	575,2 ± 47,9	567,0 ± 71,3	150,1 ± 34,4	797,1 ± 177,7	593,5 ± 86,2	142,6 ± 18,4	154,1 ± 42,3
		***	***		***	^**^	###	
G-CSF	1,0 ± 0,1	5, 9 ± 0,6	6,8 ± 0,6	0,8 ± 0,2	4,4 ± 0,8	4,7 ± 0,7	1,3 ± 0,1	0,6 ± 0,1
		***	***		***	***	###	
GM-CSF	1,5 ± 0,6	12, 9 ± 3,0	22,7 ± 4,7	0,8 ± 0,5	12,3 ± 6,3	10,5 ± 4,4	3,7 ± 1,1	1,6 ± 1,6
		*	***				##	
GRO/KC	6,8 ± 0,7	43,0 ± 2,7	41,1 ± 2,3	5,3 ± 1,0	35,5 ± 4,4	48,7 ± 8,2	9,6 ± 0,8	4,8 ± 1,1
		***	***		***	***	###	
IL-1a	21,1 ± 3,4	121,0 ± 14,9	163,6 ± 13,9	13,0 ± 3,9	106,6 ± 23,4	94,2 ± 20,8	29,9 ± 3,7	4,8 ± 2,0
		***	***		***	^**^	###	
IL-1b	5,0 ± 1,1	27,9 ± 4,4	43,7 ± 5,0	2,8 ± 1,2	24,7 ± 8,1	23,7 ± 6,5	7,7 ± 1,3	2,9 ± 2,4
		***	***		^**^	^**^	###	
MCP-1	182,9 ± 13,1	946,8 ± 120,5	739,6 ± 90,1	213,8 ± 12,3	1031,5 ± 103,1	1180,5 ± 57,3	237,2 ± 18,0	256,9 ± 22,5
		***	***		^***^	^***^	###	
IL-2	100,5 ± 21,3	671,7 ± 105,1	906,7 ± 114,3	60,2 ± 26,2	509,8 ± 147,6	493,3 ± 118,4	152,3 ± 23,1	13,7 ± 9,1
		***	***		^**^	^**^	###	
IL-5	105,4 ± 11,1	502,2 ± 58,9	593,3 ± 23,4	81,1 ± 15,5	468,2 ± 43,1	419,8 ± 75,2	130,2 ± 6,9	56,3 ± 10,3
		***	***		***	***	###	
Il-7	6,9 ± 1,3	41,3 ± 5,1	55,4 ± 5,7	4,1 ± 1,5	33,4 ± 8,8	34,7 ± 8,1	10,5 ± 1,5	3,0 ± 1,7
		***	***		^**^	***	###	
IL-12p70	8,7 ± 1,6	50,9 ± 7,6	71,9 ± 6,7	5,1 ± 2,0	38,1 ± 10,7	41,2 ± 10,0	12,5 ± 1,9	1,3 ± 0,8
		***	***		^**^	^**^	###	
IL-13	30,5 ± 9,5	219,8 ± 40,3	281,6 ± 52,2	19,4 ± 9,5	182,7 ± 65,9	173 ± 36,9	47,2 ± 9,3	3,4 ± 3,4
		***	***		^*^	^*^	#	
IL-17a	3,0 ± 0,8	21,3 ± 4,1	33,7 ± 5,2	1,4 ± 0,8	13,0 ± 5,3	18,2 ± 5,6	4,9 ± 1,2	0,0 ± 0,0
		**	***			^**^	###	
Leptin	4,9 ± 1,2	40,1 ± 14,7	40,1 ± 5,8	5,0 ± 1,1	7,9 ± 4,1	34,9 ± 10,6	7,5 ± 2,7	6,6 ± 2,4
		*	*			^***^	##	
M-CSF	2,5 ± 0,4	15,6 ± 1,6	19,6 ± 2,6	1,5 ± 0,4	11,1 ± 2,8	13,9 ± 2,4	3,6 ± 0,4	0,9 ± 0,2
		***	***		^**^	^***^	###	
MIP-2	2,5 ± 0,6	10,0 ± 3,5	15,8 ± 3,8	4,0 ± 0,8	14,6 ± 3,6	13,7 ± 4,0	2,1 ± 0,6	1,1 ± 0,5
			**		^**^	^*^		
MIP-3a	7,7 ± 0,6	48,1 ± 3,0	43,6 ± 3,8	5,0 ± 0,6	30,7 ± 4,2	50,9 ± 5,4	9,1 ± 0,7	3,8 ± 0,3
		***	***		***	***	###	
RANTES	41,9 ± 2,7	193,6 ± 8,4	208,3 ± 15,4	43,0 ± 4,2	212,2 ± 10,2	200,1 ± 18,3	53,4 ± 2,2	37,6 ± 2,9
		***	***		***	***	###	
VEGF	5,2 ± 1,6	19,3 ± 6,8	61,4 ± 14,4	0,7 ± 0,6	29,4 ± 13,9	21,9 ± 10,6	8,6 ± 3,6	0,1 ± 0,1
			***				##	

Data are presented as Mean ± SEM.

* *p* ≤ 0,05, **p ≤ 0.005, ***p ≤ 0,0005—differences within the group relative to day 0, according to Repeated Measures ANOVA, Dunnett’s Multiple Comparison Test.

# *p* ≤ 0,05, ##*p* ≤ 0.005, ###*p* ≤ 0,0005 regarding control (LPS + HVL) on the same day according to Mann-Whitney U test.

§ - baseline was measured in nine animals from each group due to technical reasons.

#### 3.2.1 Hematological and biochemical analyzes

Conducted hematological and biochemical studies on the eighth day of the experiment showed no differences in the studied blood parameters between the groups (data not shown).

#### 3.2.2 Histological analysis

**FIGURE 5:**
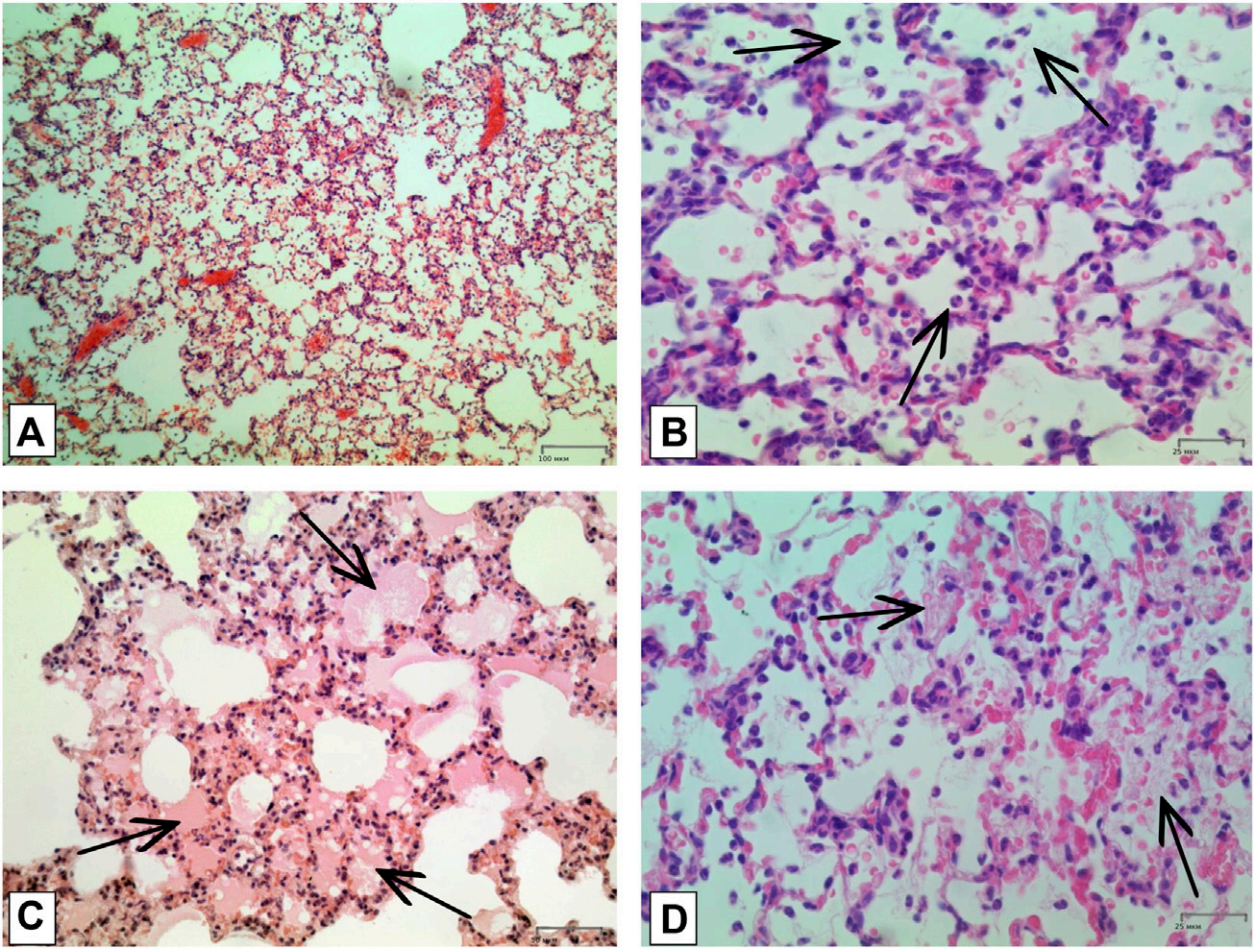
Histologic examination of lungs tissue in 4 h after LPS + HVL applied. **(A)** Interstitial and intra-alveolar infiltrates by leucocytes and erythrocytes. Magnification х 100; **(B)** Intra-alveolar infiltrates by segmento-nuclear (segmented) neutrophils. Magnification х 400; **(C)** Intra-alveolar edema. Magnification х 200; **(D)** Hyaline membranes. Magnification х 400. All described damages are indicated by black arrows.

**FIGURE 6:**
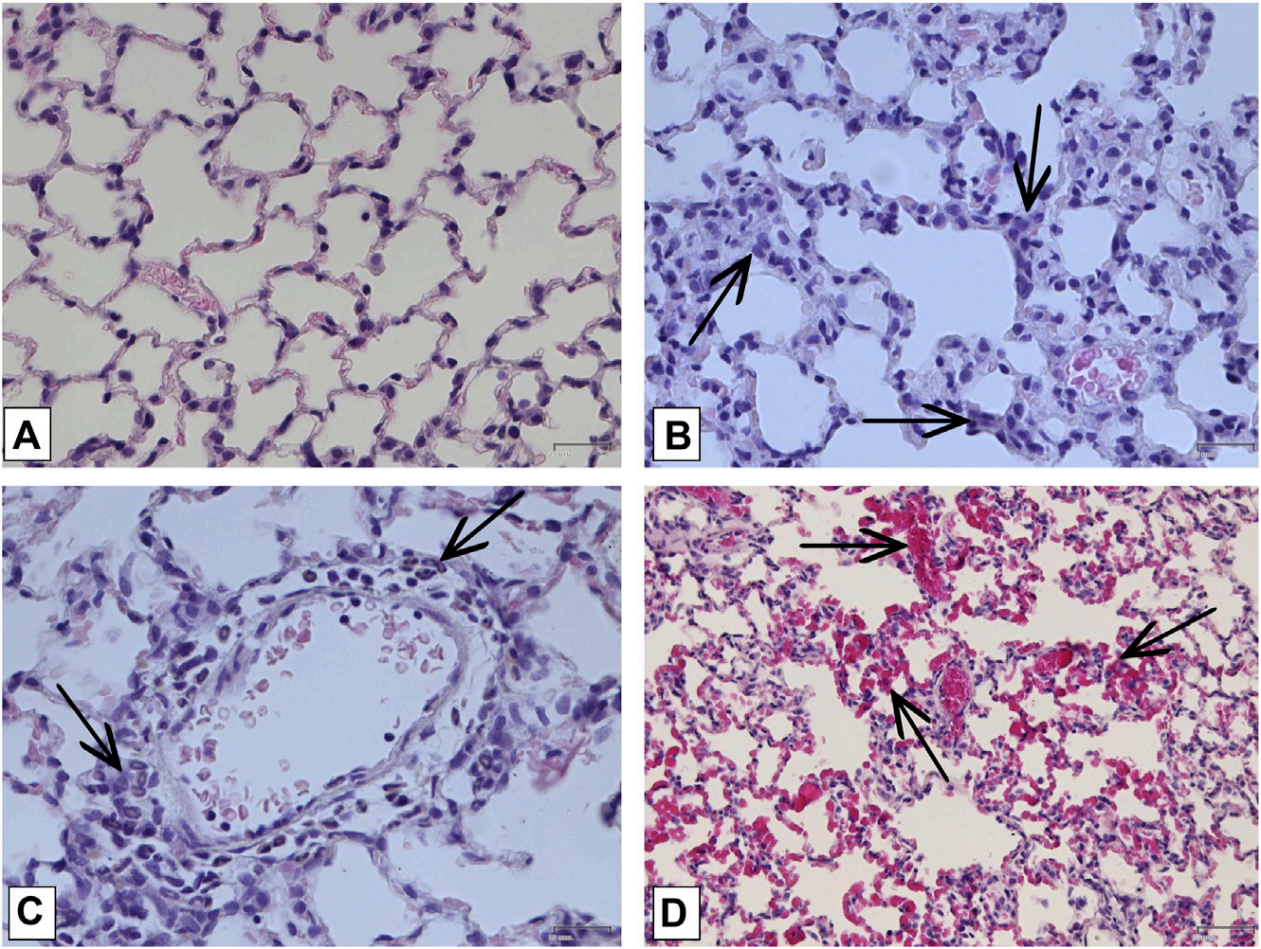
Histologic examination of lungs on the 8-th day of study. **(A)** Lung of intact animal. Magnification х400; **(B)** Lymphomacrophagal infiltration of the interstitial space Magnification х400; **(C)** Perivascular infiltration by neutrophils Magnification х 400; **(D)** Capillary fullness Magnification х 200. All described damages are indicated by black arrows.

In order to confirm the validity of the used model of ALI, three male SD rats were intratracheally injected with LPS followed by hyperventilation of the lungs, similarly as it was done for all other animals. These three SD rats were euthanized 4 h later, lungs were harvested and histologically analyzed for them. According to the results of microscopic examination, the animals showed characteristic signs of ALI. Pathological changes were characterized by interstitial perivascular edema, intraalveolar and interstitial neutrophilic infiltration, alveolar hemorrhage, and formation of hyaline membranes (Figure 5). Microscopic examination of the lungs of rats on the eighth day of the study revealed plethora of alveolar capillaries, perivascular infiltrates with lymph-macrophage cells, an increased content of macrophages in the interstitial space, infiltration with lymph-macrophage cells with a predominance of macrophages in the interstitial space (Figure 6). These changes were found during histological examination of the left lobe, right caudal lobe, right cranial, middle and accessory lobes of the lungs. It was shown that the administration of the Derinat^®^ drug significantly increases the number of perivascular infiltrates of lympho-macrophage cells, and also significantly increases the infiltration of lympho-macrophage cells with a predominance of macrophages in the interstitial space (Table 2).

**TABLE 2:** The main pathomorphological changes in the lungs on the eighth day of the study.

Drug	LPS + HVL	LPS + HVL + Derinat^®^
	N = 10	N = 10
Description of changes	The severity of changes (total score)	
Congestion of the alveolar capillaries	34	39
Hemorrhage (recent)	0	0
Perivascular infiltrates of lympho-macrophage cells	7	20
Perivascular infiltrates of neutrophils in combination with lympho-macrophage cells	17	23
Increased content of macrophages in the interstitial space	56	60
Infiltration by lympho-macrophage cells with a predominance of macrophages in the interstitial space	3	23

## 4 Discussion

There are a number of ALI models. Intratracheal administration of LPS at various doses from 0.5 to 10 mg/kg (Hong et al., 2010; Zhou et al., 2021; Li et al., 2020; Chernov et al. hyperventilation, characterized by an increase in tidal volume up to 30–120 ml/kg (Zhou et al., 2022), as well as a combination of these two factors, namely intrapulmonary through a nebulizer (Cherpanath Thomas et al., 2015), intratracheal (Bermudez et al., 2022) administration of LPS into doses of 1–2 mg/kg in combination with HVL with an increased tidal volume of 20 ml/kg. The model with HVL separately gives an objective picture of ALI only with a strong increase in tidal volume, and the model with the introduction of LPS alone gives a clinical picture of ALI only when using large doses, which leads to pathological changes incompatible with life, which do not allow long-term studies in dynamics. Combined the use of LPS + HVL can also lead to the death of animals, when LPS is administered not intratracheally, but by inhalation through a nebulizer (Cherpanath Thomas et al., 2015). For the above reasons, we chose a combination of intratracheal administration of LPS with hyperventilation of the lungs with intratracheal administration of LPS at a dose of 1 mg/kg and subsequent HVL with an increased tidal volume of 30 ml/kg to model ALI. The success of the simulation in our study was confirmed in three animals in which a histological evaluation of the lungs was performed 4 h after the simulation, which showed a pronounced pattern of lung damage. This study showed a change in the level of cytokines in the blood plasma of rats after a single injection of Derinat^®^, and also demonstrated functional changes in the respiratory system and changes in cytokines against the background of intratracheal administration of LPS + HVL. In addition, it was shown that the combination of preventive and therapeutic administration of Derinat^®^ to rats contributes to the normalization of the functional parameters of the respiratory system and a decrease in the level of cytokines in the blood plasma to the initial values already 48 h after the modeling of ALI. As a rule, doses of LPS significantly higher than 1 mg/kg are used to model ALI. For example, a dose of 5 mg/kg causes irreversible pathological changes in the lungs that are incompatible with life (Liu et al., 2020). Such an approach, obviously, does not allow a full assessment of the effect of the drug on the long-term development of the inflammatory process. The ALI model we used allowed us to save the life of animals, achieve physiological changes in the respiratory system, and made it possible to assess the condition of the animals during a week-long experiment. The presence of neutrophilic infiltrates, signs of alveolar hemorrhage and edema of lung tissues in samples from animals of the additional group, obtained 4 h after exposure to LPS + HVL, suggest that we managed to achieve changes in the lungs characteristic of ALI, as was also shown elsewhere (Li et al., 2014; Liu et al., 2018). Another sign of an acute inflammatory response is the abrupt release of interleukins from cells into the external environment, reaching a maximum within 24–48 h after antigen stimulation (Chu et al., 2016; Song et al., 2017; Liu et al., 2018; Li et al., 2020). The signs of ALI found by us correspond to the recommendations An Official American Thoracic Society Workshop Report: Features and Measurements of Experimental Acute Lung Injury in Animals (Matute-Bello et al., 2011). Our analysis of the production of cytokines in the blood plasma of mice showed that a single intratracheal administration of LPS at a dose of 1 mg/kg followed by hyperventilation of the lungs led to a sharp increase in the concentration of cytokines 24 h after exposure (on average, by an order of magnitude). The dynamics of changes in the level of cytokines in plasma showed that 48 h after exposure to LPS + HVL, the concentration of cytokines continued to increase. Derinat^®^ contains terminating nucleotide motif CpG (Filatov et al., 2013), which is a TLR9 agonist, resulting in activation of the innate immune response. Data obtained in vitro studies suggest a TLR9-mediated mechanism of immunotropic effects of drugs on based on nucleic acids, including Derinat^®^ (Filatov et al., 2013). A similar profile of an increase in the concentration of cytokines with the introduction of Derinat^®^ and LPS is explained by the fact that LPS also induces an inflammatory process through the Toll-like receptor, but only TLR4 (Martin and Wurfel, 2008; Rittirsch et al., 2008; Switalla et al., 2010). In a study by Lampe et al. evaluated the combined potential of agonists of the Toll-like receptors TLR4 and TLR9, resulting in conflicting data regarding the effect on cytokine levels and cellular infiltrates in the lungs or bronchoalveolar lavage, compared with animals vaccinated with one of the agonists. For example, Lampe et al. investigated the adjuvant potential of MPL, (a TLR4 agonist that mimics LPS) in combination with the CpG agonist TLR9 (short synthetic single-stranded DNA oligonucleotides containing unmethylated CpG motifs) and found that the combined use of CpG + MPL (TLR9 + TLR4) increases the level of pro-inflammatory and anti-inflammatory cytokines within 24 h of exposure (Lampe et al., 2020). Another study showed the joint participation of TLR9 + TLR4 in the immune response to a model of lung injury induced by sepsis in knockout animals for the corresponding gene, where it was shown that the shutdown of the corresponding genes responsible for the expression of receptors reduces the immune response in response to septic lung injury (Chen et al., 2019). In general, a number of studies have shown that the TLR group is responsible for a pronounced immune response, which consists in the release of cytokines and the development of an inflammatory response in response to infection with bacterial or viral agents. The analysis of the level of cytokines carried out in our study after exposure to LPS + HVL against the background of Derinat^®^ did not reveal a mutual enhancement of the immune response; in addition, 48 h after the modeling of ALI, the concentration of cytokines in the blood plasma returned to the values recorded in intact animals. This makes it possible to say that Derinat^®^ has immunomodulatory properties. Although there was an increase in cytokine production with additional exposure to LPS + HVL against the background of Derinat^®^, in animals of this group, a significant increase in the number of perivascular infiltrates of lymph-macrophage cells was noted, as well as infiltration of lymph-macrophage cells of the interstitial space 7 days after LPS + HVL and against the background of course application drug Derinat^®^. It can be assumed that this effect is associated with the influence of the combined effect of TLR9 + TLR4 agonists on the migration and proliferation of AP cells (Root-Bernstein 2021). Fomicheva et al. it was shown that the preventive administration of Derinat^®^ at a dose of 10 mg/kg stimulates an increase in the level of testosterone and corticosterone in animals, against the background of cold stress, immobilization and the introduction of LPS, led to an increase and subsequent maintenance of a high level of these hormones for 24 h after the stressful impacts (Fomicheva et al., 2010). Maintaining high levels of testosterone and corticosterone is an effective mechanism for increasing the body’s resistance to stress (Sapolsky et al., 2000). A number of studies have shown that corticosteroids, when administered to animals with ALI, exhibited anti-inflammatory activity, providing a protective function (Yan et al., 2017; Yang et al., 2020). The improvement in external respiration parameters in animals treated with Derinat^®^, observed in our study on the third day of the study in comparison with animals without treatment, is probably due to the anti-inflammatory effect of Derinat^®^, which may be due to stimulation of the hypothalamus-pituitary-adrenal system, and resulting in increased production of corticosteroids (Fomicheva et al., 2010). 8 days after the modeling of ALI, microscopic analysis showed that the administration of Derinat^®^ significantly increases the number of perivascular infiltrates of lymph-macrophage cells, as well as infiltration by lymph-macrophage cells with a predominance of macrophages of the alveolar space, which can be explained by the stimulating activity of Derinat^®^ in relation to cells of the innate immune response. At the same time, we found no changes in hematological and biochemical parameters in animals at the end of the study. The respiratory function and the level of cytokines in the plasma were normal. Probably, such a condition indicates a local mobilization of the immune system, which does not entail negative consequences for the organism.

## 5 Conclusion

The data obtained indicate that Derinat^®^ has pronounced immunomodulatory and anti-inflammatory properties and can be used for the treatment and prevention of acute bacterial or viral lung injury.

## Data Availability

The original contributions presented in the study are included in the article/supplementary material further inquiries can be directed to the corresponding author.
